# Hyperoxia Disrupts Lung Lymphatic Homeostasis in Neonatal Mice

**DOI:** 10.3390/antiox12030620

**Published:** 2023-03-02

**Authors:** Nithyapriya Shankar, Shyam Thapa, Amrit Kumar Shrestha, Poonam Sarkar, M. Waleed Gaber, Roberto Barrios, Binoy Shivanna

**Affiliations:** 1 Division of Neonatology, Department of Pediatrics, Texas Children’s Hospital, Baylor College of Medicine (BCM), Houston, TX 77030, USA; 2 Division of Hematology-Oncology, Department of Pediatrics, Texas Children’s Hospital, Baylor College of Medicine (BCM), Houston, TX 77030, USA; 3 Department of Pathology and Genomic Medicine, Houston Methodist Hospital, Houston, TX 77030, USA

**Keywords:** lymphangiogenesis, lymphatic function, human dermal lymphatic endothelial cells, hyperoxia, bronchopulmonary dysplasia

## Abstract

Inflammation causes bronchopulmonary dysplasia (BPD), a common lung disease of preterm infants. One reason this disease lacks specific therapies is the paucity of information on the mechanisms regulating inflammation in developing lungs. We address this gap by characterizing the lymphatic phenotype in an experimental BPD model because lymphatics are major regulators of immune homeostasis. We hypothesized that hyperoxia (HO), a major risk factor for experimental and human BPD, disrupts lymphatic endothelial homeostasis using neonatal mice and human dermal lymphatic endothelial cells (HDLECs). Exposure to 70% O_2_ for 24–72 h decreased the expression of *prospero homeobox 1* (*Prox1*) and *vascular endothelial growth factor c* (*Vegf-c*) and increased the expression of heme oxygenase 1 and *NAD(P)H dehydrogenase [quinone]1* in HDLECs, and reduced their tubule formation ability. Next, we determined *Prox1* and *Vegf-c* mRNA levels on postnatal days (P) 7 and 14 in neonatal murine lungs. The mRNA levels of these genes increased from P7 to P14, and 70% O_2_ exposure for 14 d (HO) attenuated this physiological increase in pro-lymphatic factors. Further, HO exposure decreased VEGFR3^+^ and podoplanin^+^ lymphatic vessel density and lymphatic function in neonatal murine lungs. Collectively, our results validate the hypothesis that HO disrupts lymphatic endothelial homeostasis.

## 1. Introduction

Bronchopulmonary dysplasia (BPD) is a chronic lung disease of infants characterized by interrupted alveolar and pulmonary vascular development from an imbalance between injury and repair in the developing lung [1], resulting in alveolar and pulmonary vascular simplification [2,3]. Affecting 18,000 infants annually in the United States, BPD incidence remains high in extremely premature infants [4]. BPD also has a significant economic impact because of the exorbitant costs required to provide comprehensive multidisciplinary care for these infants [5,6]. Additionally, the cardiorespiratory and neurologically morbidities associated with BPD persist into adulthood [7,8,9,10,11]. A better understanding of the major drivers that contribute to disease pathogenesis may aid in alleviating the BPD burden described above, and our study is designed to meet this need.

Inflammation is a necessary response to protect tissues against harmful stimuli and promote tissue repair; uncontrolled inflammation disrupts immune homeostasis and leads to several inflammation-mediated disorders [12], including BPD in preterm infants [13,14,15]. Inflammatory stimuli such as infection, mechanical ventilation, and hyperoxia disrupt growth factor signaling, extracellular matrix assembly, and cell proliferation in the developing lungs and contribute to BPD pathogenesis [16,17,18,19]. Systemic inflammation precedes clinical symptoms of BPD in preterm infants [20], supporting the notion that a therapeutic window of opportunity exists during the early phase of the disease to target the molecular mechanisms causing lung inflammation and its consequent deleterious effects. However, the molecular mechanisms that lead to uncontrolled inflammation remain poorly understood. Our investigation attempts to address this gap.

Originating from the embryonic veins, the lymphatic vessels develop in parallel with blood vessels. The lymphatic vascular system is a closed system of endothelial cell lined vessels that transports interstitial fluid, leukocytes, and macromolecules in a unidirectional fashion from the tissues to the draining lymph nodes, and from the latter back to the venous side of the systemic circulation. The maintenance of fluid homeostasis and regulation of immune response are well-established functions of the lymphatic system. Pre-clinical studies indicate that disrupted development and dysfunction of lymphatic vasculature causes persistent inflammation and injury in several organs, including intestines, skin, cornea, and lungs [21,22,23,24]. Further, altered growth, distribution, and function of lung lymphatics are seen in pre-clinical models and patients with chronic inflammatory lung disorders such as interstitial lung disease [21,25,26,27], asthma [23,28,29,30], and chronic obstructive pulmonary disease [31,32] whose pathophysiology is similar to BPD. However, the role of lymphatics in BPD pathogenesis is unclear. Thus, we performed in vitro and in vivo experiments to elucidate the effects of hyperoxia (HO), a well-known insult to cause experimental and clinical BPD [18,33,34,35], on human and murine lymphatic endothelial homeostasis. We used our established in vitro [36,37] and in vivo [35] HO models to test the hypothesis that HO exposure disrupts lymphatic endothelial homeostasis.

## 2. Materials and Methods

### 2.1. In Vitro Experiments

#### 2.1.1. Cell Culture

Human dermal lymphatic endothelial cells (HDLECs) were obtained from Lonza (Lonza, Morrisville, NC, USA; CC-2543, Lot no.—19TL136480) and grown in 21% O_2_ and 5% CO_2_ at 37 °C, in endothelial cell medium, as per the manufacturer’s recommendations. We used cells between passages 5 and 10 for our studies.

#### 2.1.2. Exposure of Cells to Hyperoxia

HDLECs were exposed to HO (70% O_2_ and 5% CO_2_) using a ProOx110 Compact O_2_ Controller (BioSpherix, Parish, NY, USA) for up to 72 h, as described previously [36].

#### 2.1.3. Real-Time RT-PCR Assays

RNA was extracted from cells exposed to normoxia (NO) (21% O_2_ and 5% CO_2_) or HO (70% O_2_ and 5% CO_2_) for up to 72 h, as described previously [38]. The RNA was later transcribed to cDNA and probed using the following TaqMan gene specific primers: *Heme oxygenase 1* (*HO1; Hs01110250_m1*); *NAD(P)H dehydrogenase [quinone]1* (*NQO1; Hs01045993_g1*), *prospero homeobox 1* (*Prox1*; Hs00896294_m1), *vascular endothelial growth factor-c* (*Vegf-c;* Hs01099203_m1); and *glyceraldehyde 3-phosphate dehydrogenase* (*GAPDH*; Hs02786624_g1).

#### 2.1.4. Tubule Formation Assay

We used Matrigel assay to determine HDLEC tubule formation [39,40]. HDLECs exposed to NO (21% O_2_ and 5% CO_2_) or HO (70% O_2_ and 5% CO_2_) for up to 72 h were grown in 96-well plates containing growth factor-reduced Matrigel (Corning, New York, NY, USA; 356230) at a density of 14 × 10^3^ cells per well. We used Image J software (version 1.8; https://imagej.nih.gov (accessed on 1 December 2022, 28 January 2023, and 31 January 2023); National Institutes of Health, Bethesda, MD, USA) to quantify tubule number following an 18 h incubation period in Matrigel.

#### 2.1.5. Statistical Analyses

We analyzed the results using the GraphPad Prism 9 software (GraphPad Software, La Jolla, CA, USA). Cells grown under normoxic (NO) conditions (21% O_2_ and 5% CO_2_) were used as controls, and cells exposed to HO were compared to cells grown under NO conditions. Data were tested for normality of distribution using the Shapiro–Wilk test. Data are expressed as the median with the range. The effects of HO on HDLEC tubule formation and expression of antioxidant enzymes and lymphangiogenic molecules were determined by a parametric test, such as the *t*-test for normally distributed data, and by a nonparametric test, such as the Mann–Whitney test for data that failed the normality test. A *p* value of <0.05 was considered significant.

### 2.2. In Vivo Experiments

#### 2.2.1. Animals

This study was approved and conducted in strict accordance with the federal guidelines for the humane care and use of laboratory animals by the Institutional Animal Care and Use Committee of Baylor College of Medicine (Protocol # AN-5631). C57BL/6J wild type (WT) mice (stock# 000664) were obtained from The Jackson Laboratory (Bar Harbor, ME, USA). Timed-pregnant mice raised in our animal facility were used for the experiments. 

#### 2.2.2. Hyperoxia Experiments

The HO exposure experiments were conducted in sealed plexiglass chambers into which 70% blended oxygen was continuously circulated, as described before [41]. The WT mice either remained in NO (21% O_2_) or were exposed to HO (70% O_2_) from postnatal day (P) 1 to 14. We avoided oxygen toxicity in the dams by rotating them between NO- and HO-exposed litters every 24 h. 

#### 2.2.3. Analyses of Lung Lymphatic Vascularization

We harvested the lung tissues to quantify lymphatic vascularization on P7 (*n* = 3/group; *n* = 1 male, 2 females in the normoxia group and 2 males, 1 female in the hyperoxia group)) and P14 (*n* = 4/group; *n* = 1 male, 3 females in the normoxia group and 2 males, 2 females in the hyperoxia group). Briefly, the lungs were inflated with 4% paraformaldehyde at a pressure of 25 cm H_2_O for at least 10 min, fixed with 4% paraformaldehyde for 18 h, and washed with and stored in 70% ethanol at 4 °C until embedding with paraffin. The paraffin-embedded lung sections were sliced into 5µm thin sections using a microtome and subjected to immunofluorescence studies. We deparaffinized sections from paraffin-embedded lung tissues in xylene and graded alcohols before subjecting them to antigen retrieval by boiling at 100 °C in EDTA. We initially blocked endogenous peroxidase activity and nonspecific protein binding by incubating the lung sections with 3% H_2_O_2_ and 10% normal donkey serum, respectively, for 1 h. Next, we incubated the lung sections overnight at 4 °C with the following primary antibodies: anti-PDPN (Developmental Studies Hybridoma Bank, University of Iowa, Iowa city, Iowa; 8.1.1, dilution 1:400) and anti-VEGFR3 (R and D systems, Inc., Minneapolis, MN; AF743, dilution 1:200). We detected the primary antibodies by incubation with fluorescein–conjugated goat anti-Syrian hamster (Alexa Fluor 488, Invitrogen; Eugene, OR; A-21110, dilution 1:300) and donkey anti-goat (Alexa Fluor 594, Invitrogen; Eugene, OR; A-11058, dilution 1:300) secondary antibodies for 2 h in the dark. All the slides were counterstained with 4′,6-diamidino-2-phenylindole (DAPI) and analyzed using the Keyence microscope (Keyence Corporation, Itasca, IL, USA). We examined at least 10 random nonoverlapping fields of 20X PDPN- and VEGFR3-stained images from both the right and left lungs of each animal to quantify lymphatic vascularization. The lung lymphatic density was estimated by normalizing the number of lymphatic vessels to the lung surface area using Image J, as described before [26].

#### 2.2.4. Lung Tissue Extraction and Real-Time RT-PCR Assays

The lungs were snap-frozen in liquid nitrogen on P7 (*n* = 3/group; *n* = 1 male, 2 females in the normoxia group and 2 males, 1 female in the hyperoxia group) and P14 (*n* = 3/group; *n* = 2 males, 1 female in the normoxia group and 2 males, 1 female in the hyperoxia group) and stored at −80 °C for subsequent RNA studies. Total lung RNA was extracted and reverse transcribed to cDNA [42] and probed using the following TaqMan gene specific primers: *prospero homeobox 1* (*Prox1*; Mm00435969_m1), *vascular endothelial growth factor-c* (*Vegf-c;* Mm00437310_m1), and *glyceraldehyde 3-phosphate dehydrogenase* (*GAPDH*; Mm99999915_g1).

#### 2.2.5. Analyses of Lung Lymphatic Function

We studied the lymphatic function by measuring the pulmonary lymphatic flow, as described recently [43]. Briefly, WT mice were exposed to NO or HO from P1 to P14, as described above. The HO-exposed mice were allowed to recover in NO conditions from P15 to P21. On P21, NO- and HO-exposed mice anesthetized with 2% isoflurane were administered 35 μL of 5 mg/mL AlexaFluor568-labelled dextran (10,000 kD MW, Invitrogen; D-22912) intratracheally. Fifty minutes after administering the Fluor-labelled dextran, we harvested the mediastinal lymph nodes (mLNs) and measured their fluorescent intensity. 

#### 2.2.6. Statistical Analyses

GraphPad Prism 9 software was used to analyze the results. Animals reared under the routine housing conditions with exposure to room air (normoxia (NO), 21% oxygen) were used as controls. The HO-exposed animals were compared with NO-exposed animals. Data were tested for normality of distribution using the Shapiro–Wilk test. Data are expressed as the median with the range. The interaction between time and HO exposure on the expression of the lymphangiogenic molecules was determined by ANOVA if the data were normally distributed or by the Kruskal–Wallis test for those that failed the normality test. The effects of HO exposure on the density and function of lymphatics were determined by the *t*-test if the data were normally distributed or by the Mann–Whitney test for those that failed the normality test. A *p* value of <0.05 was considered significant.

## 3. Results

**Figure 1:**
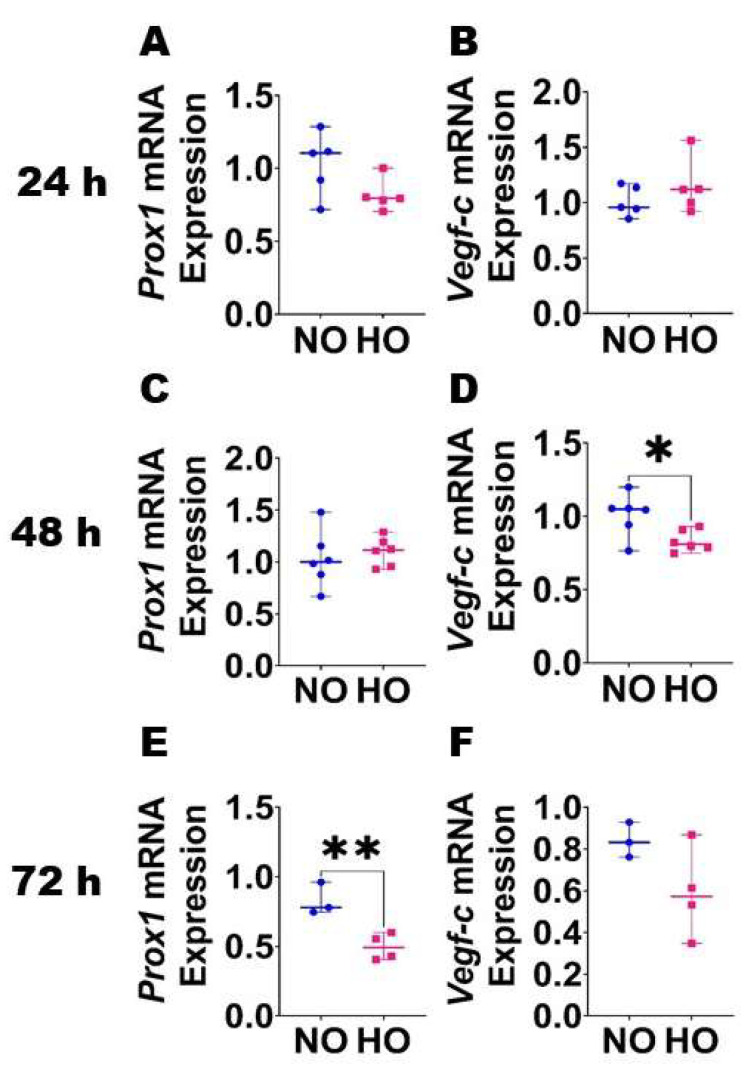
Effects of HO exposure on the expression of lymphangiogenic molecules in HDLECs. RNA was extracted at the indicated time points from cells grown on six-well plates to 60–70% confluence and exposed to NO (21% O_2_ and 5% CO_2_) or HO (70% O_2_ and 5% CO_2_) for up to 72 h. The RNA was later transcribed to cDNA and subjected to real-time RT-PCR analysis to quantify the mRNA levels of *Prox1* (**A**,**C**,**E**) and *Vegf-c* (**B**,**D**,**F**) at 24 h (**A**,**B**), 48 h (**C**,**D**), and 72 h (**E**,**F**). Values represent the median with range (*n* = 3–6/group). Significant differences between exposures are indicated by *, *p* < 0.05, **, *p* < 0.01 (*t*-test).

Expression of lymphangiogenic molecules in HDLECs following HO exposure: To determine the effects of HO on lymphatic endothelial homeostasis, we quantified the mRNA levels of prolymangiogenic molecules, *Prox1* and *Vegf-c*, in HO-exposed HDLECs. HO exposure had a time-dependent effect on the expression of these pro-lymphatic genes. The mRNA expression of *Vegf-c* decreased after 48 h of HO exposure (Figure 1D), while the expression of *Prox1* decreased after 72 h of HO exposure (Figure 1E). 

**Figure 2:**
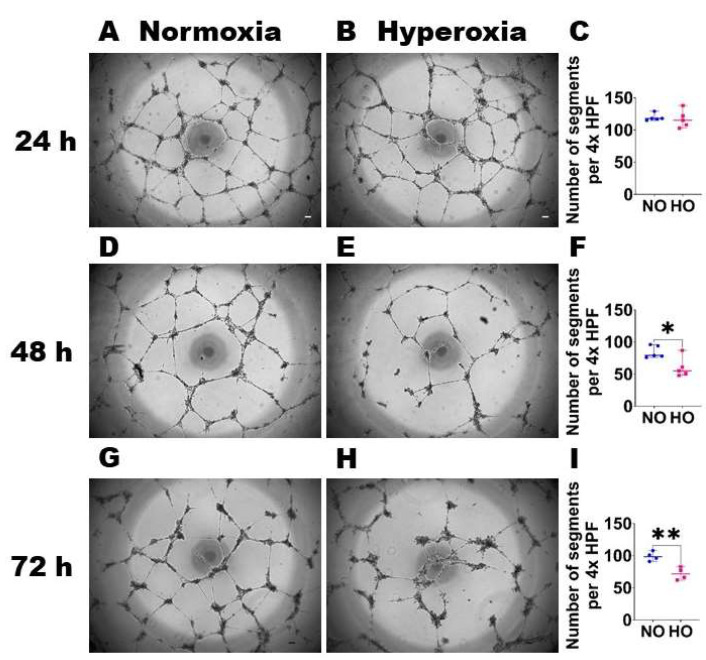
Effects of *HO* exposure on HDLEC tubule formation. Tubule formation assay was performed at the indicated time points on cells grown on six-well plates to 60% confluence and exposed to NO (21% O_2_ and 5% CO_2_) or HO (70% O_2_ and 5% CO_2_) for up to 72 h. (**A**, **B**, **D**, **E**, **G** and **H**). Representative photographs showing tubule formation of NO (**A**,**D**,**G**) and HO (**B**,**E**,**H**) exposed cells at 24 h (**A**,**B**), 48 h (**D**,**E**), and 72 h (**G**,**H**). Scale bar = 100 µm. C, F, and I. Quantification of tubule number at 24 h (**C**), 48 h (**F**), and 72 h (**I**). Values are presented as the median with the range (*n* = 4–5/group). Significant differences between exposures are indicated by *, *p* < 0.05, **, *p* < 0.01 (*t*-test).

HO exposure disrupts HDLEC tubule formation: We next determined if HO exposure disrupts lymphangiogenesis by performing the tubule formation assay on HDLECs exposed to NO or HO for up to 72 h. While the HDLEC tubule number was comparable between NO- and HO-exposed cells at 24 h (Figure 2A–C), the HDLEC tubule number was decreased in HO-exposed cells compared with NO-exposed cells at 48 h (Figure 2D–F) and 72 h (Figure 2G–I). These findings suggest that HO negatively affects lymphangiogenesis.

**Figure 3:**
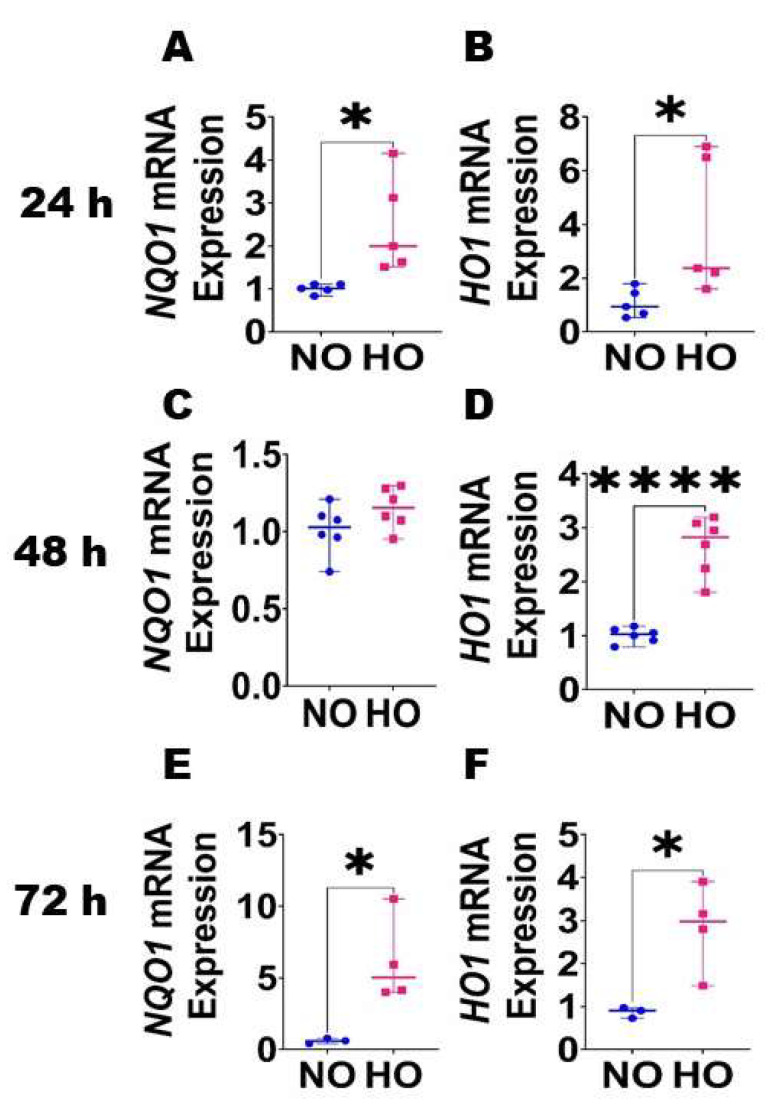
Effects of HO exposure on the expression of antioxidant enzymes in HDLECs. RNA was extracted at the indicated time points from cells grown on six-well plates to 60–70% confluence and exposed to NO (21% O_2_ and 5% CO_2_) or HO (70% O_2_ and 5% CO_2_) for up to 72 h. The RNA was later transcribed to cDNA and subjected to real-time RT-PCR analysis to quantify the mRNA levels of *NQO1* (**A**,**C**,**E**) and *HO1* (**B**,**D**,**F**) at 24 h (**A**,**B**), 48 h (**C**,**D**), and 72 h (**E**,**F**). Values represent the median with range (*n* = 3–6/group). Significant differences between exposures are indicated by *, *p* < 0.05, ****, *p* < 0.0001 (*t*-test).

Expression of antioxidant enzymes in HDLECs following HO exposure: Since oxidative stress can disrupt endothelial cell homeostasis by affecting the expression of angiogenic molecules and impairing the tubule-forming capability of these cells, we also quantified the expression of the well-known antioxidant enzymes, NQO1 and HO1, by real-time RT-PCR analysis in our in vitro model. HO affected the *NQO1* mRNA expression in a biphasic manner, with increased expression after 24 h (Figure 3A) and 72 h (Figure 3E) of exposure and no effect after 48 h (Figure 3C) of exposure compared to NO exposure for identical periods. In contrast, HO exposure increased *HO1* mRNA expression at all three-time points compared to NO exposure (Figure 3B,D,F), with a peak effect occurring at 48 h (Figure 3D).

**Figure 4:**
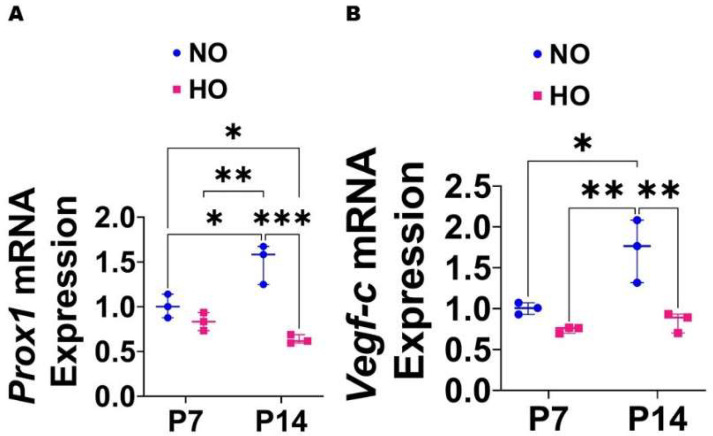
Expression of lymphangiogenic molecules in HO-exposed neonatal murine lungs. Gene expression studies were carried out by RT-PCR analyses on whole lung RNA isolates from WT mice exposed to NO (21% O_2_) or HO (70% O_2_) for 7 and 14 d. (**A**–**B**): the quantification of *Prox1* (**A**) and *Vegf-c* (**B**) mRNA levels. Values are presented as the median with the range (*n* = 3/group). Significant differences between exposures are indicated by *, *p* < 0.05, **, *p* < 0.01, and ***, *p* < 0.001 (ANOVA).

HO exposure decreases the expression of the lymphangiogenic molecules in the lungs of neonatal mice: Based on the effects of HO on the expression of lymphangiogenic molecules in HDLECs, we investigated if HO exerts a similar effect in neonatal murine lungs. The lung mRNA levels of *Prox1* and *Vegf-c* increased significantly from P7 to P14 by 1.5–fold and 1.7–fold, respectively, in mice reared in NO conditions. While the mRNA levels of these lymphangiogenic molecules were comparable between HO and NO exposed lungs on P7, the levels were significantly decreased in HO-exposed lungs on P14 (*Prox1*: HO, 0.63 ± 0.05 vs. NO, 1.5 ± 0.22; *p* < 0.001 (Figure 4A), *Vegf-c*: HO, 0.84 ± 0.12 vs. NO, 1.72 ± 0.38; *p* < 0.01 (Figure 4B)). These results suggest that HO exposure prevents the normal increase in the expression of lymphangiogenic molecules in the developing lungs.

**Figure 5:**
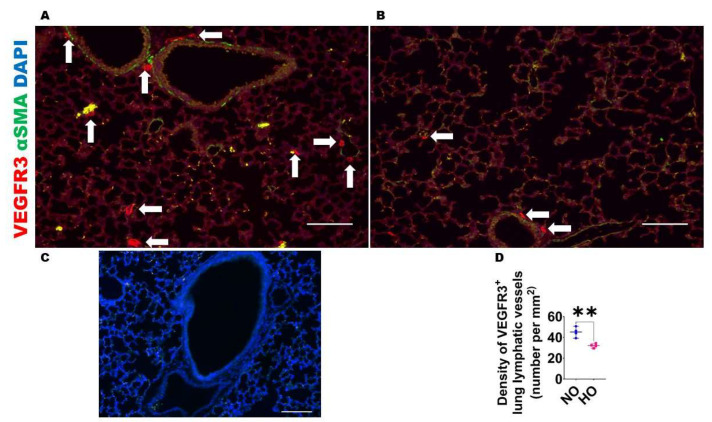
Lung VEGFR3^+^ lymphatic density in neonatal mice exposed to HO. The lung lymphatic density was determined by quantifying VEGFR3^+^ lung vessels (arrows) on P14 in WT mice exposed to 21% O_2_ (NO) or 70% O_2_ (HO) from P1 to P14. (**A**–**B**). Representative VEGFR3^+^ (red), αSMA (green), and DAPI (blue) immunofluorescence-stained lung sections from mice exposed to NO (**A**) and HO (**B**) for 14 d. (**C**). Representative lung section from NO exposed animals stained with secondary antibodies only (negative control). Scale bar = 100 µm. (**D**). Quantification of VEGFR3^+^ lung vessels. Values are presented as the median with the range (*n* = 4/group). Significant differences between exposures are indicated by **, *p* < 0.01 (*t*-test).

**Figure 6:**
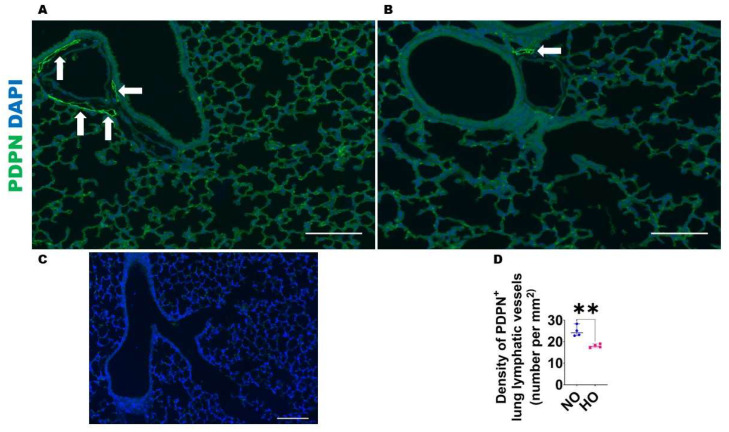
Lung PDPN^+^ lymphatic density in neonatal mice exposed to HO. The lung lymphatic density was determined by quantifying PDPN^+^ lung vessels (arrows) on P14 in WT mice exposed to 21% O_2_ (NO) or 70% O_2_ (HO) from P1 to P14. (**A**–**B**). Representative PDPN^+^ (green) and DAPI (blue) immunofluorescence-stained lung vessels from mice exposed to NO (**A**) and HO (**B**) for 14 d. (**C**). Representative lung section from NO exposed animals stained with secondary antibody only (negative control). Scale bar = 100 µm. (**D**). The quantification of PDPN^+^ lung vessels. Values are presented as the median with the range (*n* = 4/group). Significant differences between exposures are indicated by **, *p* < 0.01 (*t*-test).

**Figure 7:**
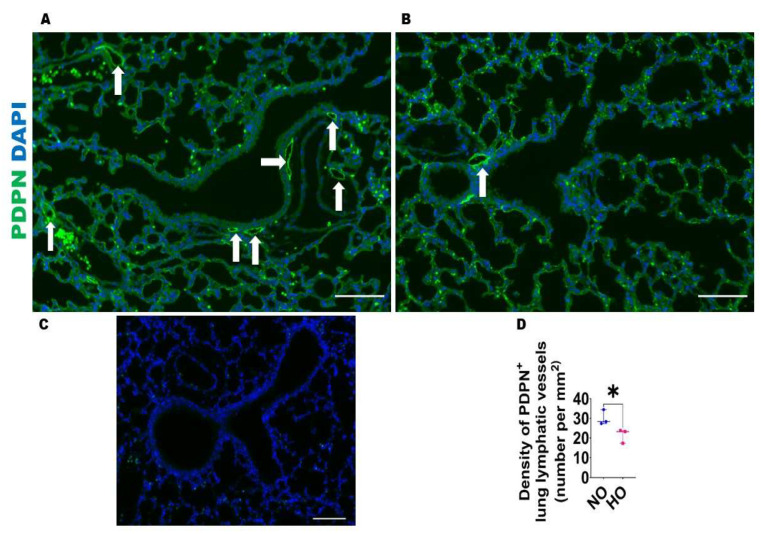
Lung PDPN^+^ lymphatic density in neonatal mice exposed to HO. The lung lymphatic density was determined by quantifying PDPN^+^ lung vessels (arrows) on P7 in WT mice exposed to 21% O_2_ (NO) or 70% O_2_ (HO) from P1 to P7. (**A**–**B**). Representative PDPN^+^ (green) and DAPI (blue) immunofluorescence-stained lung vessels from mice exposed to NO (**A**) and HO (**B**) for 7 d. (**C**). Representative lung section from NO exposed animals stained with secondary antibody only (negative control). Scale bar = 100 µm. (**D**). Quantification of PDPN^+^ lung vessels. Values are presented as the median with the range (*n* = 3/group). Significant differences between exposures are indicated by *, *p* < 0.05 (*t*-test).

HO exposure decreases lung lymphatic density in neonatal mice: Given the evidence that HO decreases the expression of lymphangiogenic molecules, we next investigated if this finding is associated with altered lung lymphatic density on P14. VEGFR3^+^ vessels were observed around larger airways and blood vessels as well in the alveolar regions (Figure 5), whereas PDPN^+^ vessels were located predominantly around larger airways and blood vessels (Figure 6). After normalizing to the lung surface area, HO-exposed mice also had decreased VEGFR3^+^ (Figure 5A–D) and PDPN^+^ (Figure 6A–D) lymphatic vessels compared with NO-exposed mice. We also observed that HO-exposed mice had reduced PDPN^+^ (Figure 7A–D) lymphatic vessels compared with NO-exposed mice at P7. These findings indicate that HO decreases lymphatic density in the developing lungs. 

**Figure 8:**
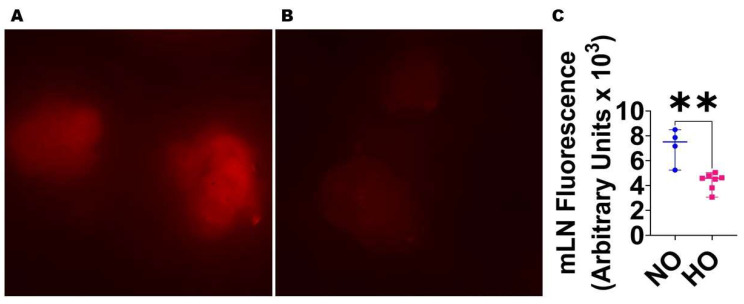
Pulmonary lymphatic flow in HO-exposed mice. The pulmonary lymphatic flow was determined by quantifying the mediastinal lymph node (mLN) uptake of dextran-568 on P21 in WT mice exposed to 21% O_2_ (NO) or 70% O_2_ (HO) from P1 to P14. (**A**–**B**). Representative fluorescence microscopic images of mLNs from mice exposed to NO (**A**) and HO (**B**) 50 min after intra-tracheal administration of dextran-568 (red). (**C**). Quantitative analysis of mLN fluorescence intensity. Values are presented as the median with the range (*n* = 4–7/group). Significant differences between exposures are indicated by **, *p* < 0.01 (*t*-test).

Neonatal HO exposure decreases lung lymphatic function in mice: Finally, we sought to investigate if the reduced lung lymphatic density is associated with decreased lymphatic function in mice exposed to neonatal HO. We demonstrate that the mLN fluorescence is lower in HO-exposed mice than in NO-exposed mice (Figure 8). 

## 4. Discussion

Herein, we studied the effects of HO exposure on human dermal lymphatic endothelial cell lymphangiogenic factor expression, tubule formation, and redox homeostasis in vitro and the consequences of HO exposure on lung lymphatic endothelial cell homeostasis in neonatal mice in vivo. Our in vitro experiments suggest that moderate HO exposure decreases the expression of prolymangiogenic molecules, increases oxidative stress, and reduces the lymphangiogenic capacity in human dermal lymphatic endothelial cells. Consistent with these in vitro findings, our in vivo experiments indicate that HO exposure decreases the expression of prolymangiogenic molecules and reduces the density and function of the lymphatics in neonatal murine lungs. 

The levels of angiogenic molecules such as VEGF, VEGFR1, VEGFR2, angiopoietins, and tyrosine-protein kinase receptor-2 (TIE-2) are decreased in human and experimental BPD [44,45,46,47,48]. However, if lymphangiogenic molecules are similarly altered in experimental and human BPD needs to be better studied. Because lymphatic and blood vasculature develop in parallel and lymphatics regulate inflammation, which is a final common mediator of BPD pathogenesis, we investigated the effects of HO exposure, a known BPD risk factor, on the lymphangiogenic molecules. We used neonatal mice and human cells to increase the translational potential of our study. Similar to its effects on the expression of angiogenic molecules, HO decreased the expression of *Prox1* and *Vegf-c* in neonatal murine lungs and in HDLECs. The mechanisms behind this time-dependent effect of HO on the expression of these genes in HDLECs are unclear and require further studies. We chose to measure these two molecules for the following reasons. Prox1 is a master nuclear transcription factor that confers the lymphatic endothelial cell fate, and in its absence, the LECs fail to differentiate and proliferate [49,50,51]. Vegf-c promotes the sprouting of initial lymphatic vessels from embryonic veins [52] and is a potential target to modulate lymphangiogenesis and lymphatic function under various pathological states [53,54,55,56,57]. Several studies show that the exposure of neonatal rats to hyperoxia decreases Prox1 expression in neuronal tissues [58,59,60]. Our finding is consistent with these studies but provides new information that a similar phenomenon occurs in the developing lungs. Additionally, we had observed a similar effect of HO on other well-known pro-lymphatic factors, such as adrenomedullin, calcitonin receptor-like receptor, and receptor activity modifying protein 2 in the developing lungs [61]. The effects of HO on Vegf-c expression are less well characterized than its effects on Prox1. In human infants, Vegf-c levels correlate directly with antenatal glucocorticoid administration and inversely with postnatal age [62]; however, whether the levels correlate with respiratory morbidities, including BPD, is unclear. In a neonatal murine model of proliferative retinopathy, exposure to 80% O_2_ for 5 days did not alter *Vegf-c* mRNA levels in the ocular tissues; however, following a period of 24 h recovery in room air after 80% O_2_ exposure, the *Vegf-c* levels increased by three-fold [63]. The recovery period following HO exposure mimics a hypoxic state secondary to HO-mediated inhibition of angiogenesis, and the cellular response to such a state is the stabilization of hypoxia-inducible factor 1 (HIF1), which is a transcription factor controlling the expression of several genes, including the VEGF family [64,65,66]. Therefore, an increase in *Vegf-c* levels following hypoxia is not completely unexpected. The lack of the immediate effect of HO on *Vegf-c* levels in the above study [63], in contrast to the inhibitory effect of HO on *Vegf-c* levels in our study, may be related to differences in the duration of oxygen exposure and the tissues being studied. The mechanisms through which HO regulates the expression of these lymphangiogenic molecules are unclear at this time point, thus warranting further studies to address this gap.

In addition to the decreased expression of angiogenic molecules, lung angiogenesis is inhibited in human [2,3,17,44,67] and experimental [4,68,69,70,71,72,73] BPD, suggesting that lymphangiogenesis may be similarly altered in this disease. Further, increased tissue leukocytes and edema are hallmarks of an inflammatory disease such as BPD, and because the lymphatic system regulates fluid homeostasis and leukocyte trafficking, it is conceivable that the lymphatic system plays a pivotal role in regulating tissue inflammation and repair in these disorders. Lung lymphatics are crucial for both survival immediately after birth [74,75] and lung health maintenance in later life [43]. Mice lacking lung lymphatics have increased neonatal mortality from respiratory failure because of a diminished clearance of interstitial fluid and inadequate lung expansion [74,75]. In contrast, lymphangiectasia, from the overexpression of lymphangiogenic factors, can also increase neonatal mortality from respiratory failure [76], indicating the need for optimal lymphatic structure and function for healthy lungs. We quantified lymphatic density and lymphatic function in the lungs to determine lung lymphatic health. mLN fluorescence is directly proportional to lung lymphatic function because lung lymphatics drain into these nodes. Therefore, we determined lung lymphatic function by quantifying mLN fluorescence intensity. Our findings indicate that insults such as HO that cause BPD inhibit lymphangiogenesis in vitro and decrease lymphatic density and function in vivo. Collectively, our data suggest that HO negatively affects lung lymphatic health. Our findings contrast that of a small cohort study comparing nine BPD patients with four control subjects, which showed that PDPN-stained alveolar lymphatic vessel density was increased in BPD patients [77]; however, the lymphatic function was not investigated in this study. Whether an insult increases or inhibits lymphangiogenesis may depend upon the nature and duration of the insult, the organ under investigation, the developmental stage of the organ, and the underlying pathophysiology. For instance, increased lymphangiogenesis is observed in the experimental models of chronic airway inflammation secondary to an infectious agent, whereas an opposite effect is observed in an experimental model of allergic airway disease [23,29,30]. Similarly, while the lymphatic density is decreased in patients with asthma [28], the converse is true in patients with chronic obstructive pulmonary disease [31,32] and idiopathic pulmonary fibrosis [21,25]. It is also possible that the lung lymphatic density and function vary with the endotype and phenotype of a complex chronic disease such as BPD.

Oxidative stress is a significant mechanistic risk factor for decreased or disrupted angiogenesis in BPD [78,79,80]. We measured the expression of the antioxidant enzymes, NQO1 and HO1, in HDLECs because they regulate the redox equilibrium in cells [81,82]. We observed *NQO1* and *HO1* to be significantly altered in our model. The molecular mechanisms behind the biphasic effect of HO on the expression of these genes in HDLECs are unclear and require further study. In addition to reflecting the oxidative stress status of a cell, *HO1* and *NQO1* have several vasculoprotective effects [83,84,85,86]. Therefore, our finding of increased *HO1* and *NQO1* expression in HDLECs suggests that these cells are under oxidative stress following HO exposure, and they upregulate *HO1* and *NQO1* expression as an adaptive response to mitigate the effects of HO. Overall, our findings suggest that HO exposure causes oxidative stress in lymphatic endothelial cells, similar to its effects on blood endothelial cells. Oxidative stress may be one of the primary drivers of disrupted lymphatic endothelial cell homeostasis following HO exposure.

There are a few limitations to our studies. We used human dermal lymphatic endothelial cells rather than human pulmonary lymphatic endothelial cells for our studies. We recognize that there may be endothelial cell type-specific phenotypic responses to HO; however, because human dermal lymphatic endothelial cells are readily available, they have been widely used to determine the mechanisms through which lymphatics regulate pathological states in various organs [87,88]. Further, HO exerted similar effects on these cells and the lymphatic vasculature in neonatal murine lungs. We also did not quantify lung lymphatic density at various regions of the lungs, i.e., airway, vascular, and alveolar lymphatic density. Nevertheless, our findings are meaningful because we show that the whole lung lymphatic density is reduced, and this quantitative deficiency is associated with decreased lymphatic function. Finally, our findings suggest an association between lymphatic insufficiency and BPD pathogenesis. The future loss and gain of function studies involving pro-lymphatic and anti-lymphatic factors are needed to illustrate the direct role of lymphatics in BPD pathogenesis.

In summary, using in vitro and in vivo models, we demonstrate that HO exposure decreases the expression of pro-lymphatic factors, increases lymphatic endothelial cell oxidative stress, and reduces lymphangiogenesis, lymphatic density, and lymphatic function. To the best of our knowledge, this is the first study to investigate the effects of HO on the lymphatic vasculature in developing lungs. Our findings provide a rationale for performing mechanistic studies targeting lymphatics in experimental models of BPD and lymphatic phenotyping studies in BPD infants.

## Data Availability

The data presented in this study are available in the article.
